# Comparisons of the energy efficiency and intraocular safety of two torsional phacoemulsification tips

**DOI:** 10.1186/s12886-022-02619-0

**Published:** 2022-10-03

**Authors:** Yan-Xiu Sun, Rong Cao, Zi-Yuan Liu, Hua-Qin Xia, Yu-Jie Cen, Lu Gao, Dan-Dan Shi

**Affiliations:** 1 Department of Ophthalmology, Key Laboratory of Restoration of Damaged Ocular Nerve, Peking University Third Hospital, Haidian District, 49 North Garden Road, BeijingBeijing, 100191 China; 2 Department of Ophthalmology, Yan’an Branch of Peking University Third Hospital, Yan’an Hospital of Traditional Chinese Medicine, Shanxi, China

**Keywords:** Intrepid® balanced tip, Kelman tip, Cumulative dissipated energy, Corneal endothelium loss rate, Central corneal thickness

## Abstract

**Background:**

During cataract phacoemulsification surgery, an Intrepid® balanced (IB) tip can achieve a larger amplitude, which may lead to higher energy efficiency than a Kelman (K) tip when paired with a torsional phaco platform. In this retrospective cohort study, we compared their energy efficiency and damage to the cornea under a new energy setting.

**Methods:**

The medical records of 104 eyes of 79 patients were reviewed, with 47 eyes belonging to the IB group and 57 eyes belonging to the K group. All surgeries were performed on an Alcon Centurion® platform with gravity infiltration. Surgical parameters, visual outcome, central corneal thickness (CCT) changes, and endothelial cell density (ECD) loss rate were recorded and calculated.

**Results:**

No significant differences in postoperative best corrected visual acuity (BCVA), intraocular pressure (IOP), total ultrasound time, estimated fluid aspirated, CCT changes, or ECD loss rate were observed between the two groups. We divided the included eyes into soft nucleus and hard nucleus subgroups and found lower cumulative dissipated energy (CDE, 8.15 ± 8.02 vs 14.82 ± 14.16, *P* = 0.023), cumulative torsional energy (CTE, 8.06 ± 7.87 vs 14.13 ± 13.02, *P* = 0.027), and cumulative longitudinal energy (CLE, 0.09 ± 0.17 vs 0.69 ± 1.37, *P* = 0.017) in the IB group than in the K group, implying less energy used and higher energy efficiency of the IB tip.

**Conclusion:**

Lower CLE in the IB group indicates fewer phaco tip obstructions and a significantly higher capability to conquer hard nuclei with IB tips with statistical significance. With an ultra-perfusion cannula, the balanced tip does not cause more corneal damage.

## Background

Senile cataracts are the leading cause of blindness worldwide. Higher energy efficiency and less tissue damage are important to ensure the ideal results of cataract phacoemulsification surgeries. New surgical equipment and techniques focus on improving handpiece efficiency and reducing corneal incision size without increasing corneal damage. A smaller incision may reduce surgically induced astigmatism but may also face the risk of insufficient infusion, which results in corneal burns.

The combination of a torsional phacoemulsification platform and a torsional handpiece can produce side-to-side rotary oscillations of the phaco tip. Torsional motion has the tip subtend an arc, with some suggesting that this is more efficient than longitudinal power, which uses a jackhammer approach to accomplish the same task [1]. The torsional phaco tip includes a shaft that has one or two bends and a cutting edge. The small-angle rotational movement of the shaft is translated into a horizontal stroke at the tip of the needle. The design of the tips not only increases the efficiency of phacoemulsification but also reduces heat generation and burns at the incision site [2].

**Fig. 1:**
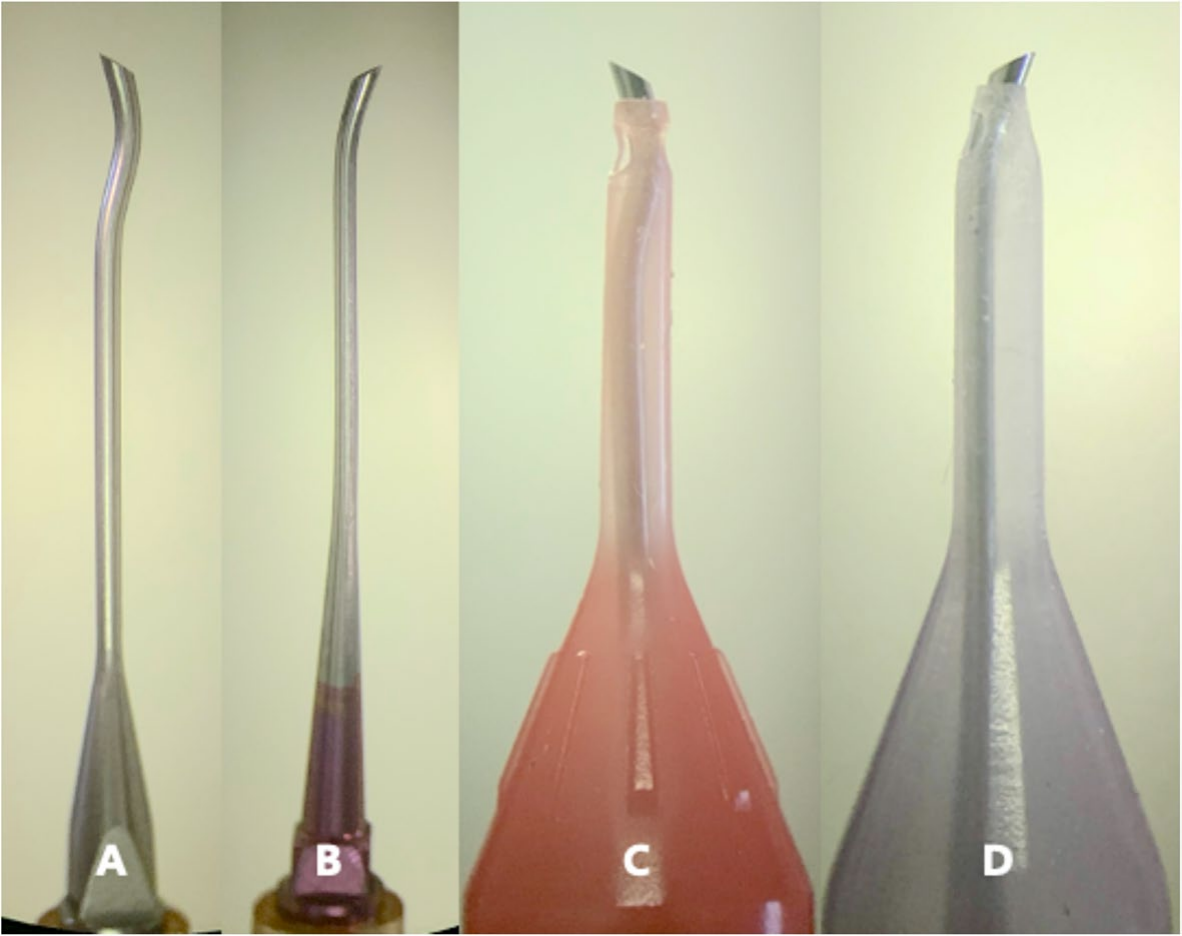
Torsional phaco-tips and the infusion cannula applied in the study. **A** 0.9 mm 45°bevel Intrepid® balanced ABS needle, **B** 0.9 mm 22-degree bend/45°bevel Kelman ABS needle, **C** Balanced tip with 0.9 mm Ultrainfusion cannula for 2.2 mm incision, **D** Kelman tip with 0.9 mm standard infusion cannula for 3.2 mm incision

The Alcon Kelman tip has a 20° bend at the distal end, with an amplitude of up to 130 μm, which can produce a strong cutting ability. With a standard infusion cannula, it is suitable for 2.75–3.2 mm incisions. The Alcon Intrepid® balanced tip matched with the same platform, and the same Ozil handpiece had double bending. The distal end of the needle can reach a larger amplitude up to 192 μm. Because the axial direction is more stable, the needle produces less vibration and further reduces heat generation [3]. With an ultrainfusion cannula, it can be applied to 2.2 mm incisions (Fig. 1). This study intends to observe the energy parameters applied during the operation on the same Alcon Centurion® silver vision system platform using the same gravity infusion mode to evaluate the difference in efficiency of the two torsional phaco tips. The postoperative corneal thickness and endothelial cell loss rate will also be observed to evaluate the damage to the cornea and the safety of both phaco tips under certain parameter sets.

## Methods

### Patients

This was a retrospective cohort study approved by the ethical committee of Peking University Third Hospital (M2021666) and conducted in accordance with the tenets of the Declaration of Helsinki. Patients with senile cataracts who underwent surgical intervention were included from June to September 2020 at Peking University Third Hospital Yan-An Hospital. The postoperative follow-up duration was at least 3 months. We excluded children, gravida, and patients with other vision-threatening disorders, such as glaucoma, macular edema, and diabetic retinopathy. Patients who did not complete the follow-up program were excluded. Written informed consent for using medical records was obtained from each patient.

### Surgical parameters

Forty-seven eyes of the Intrepid® balance tip (IB) group and 57 eyes of the Kelman tip (K) group were enrolled. Surgeries were performed by one experienced ophthalmologist. All surgeries were performed using an Alcon Centurion® silver vision system (Alcon Surgical, Fort Worth, Texas, USA) under gravity infiltration mode with an Alcon Ozil handpiece. A 0.9 mm 45-degree bevel IB aspiration bypass system (ABS) needle (Alcon Surgical, Fort Worth, Texas, USA) and a 0.9 mm Ultra infusion cannula that matched the 2.2 mm main incision were applied in the IB group (Fig. 1, A and C). A 0.9 mm 22-degree bend/45-degree bevel Kelman ABS needle (Alcon Surgical, Fort Worth, Texas, USA) and a 0.9 mm standard infusion cannula that matched the 3.2 mm main incision were used in the K group (Fig. 1, B and D). The energy settings were as follows: longitudinal power closed, torsional ultrasound (US) linear from 50 to 85% in the K group and 30% to 60% in the IB group. During phaco, the bottle height was set to 104 cmH_2_O, vacuum was set linearly from 250 to 475 mmHg, and aspiration flow was set linearly from 30 cc/min to 40 cc/min. Intelligent phaco (IP) mode was used in both groups. Pulse mode was activated when negative pressure reached 95% of the predetermined value, and every pulse lasted 7 ms with 100 ms of intermittent time. For each pulse, the ratio of longitudinal power and torsional power was 0.7, and the cumulative time of longitudinal US was no more than 200 ms.

A clear corneal incision was made on the steep axis with a 2.2 or 3.2 mm dual-bevel metal keratome blade (Alcon Surgical, Inc.). A viscoelastic device was used to stabilize the anterior chamber. A 5.0 to 5.5 mm continuous curvilinear capsulorhexis was made. One-piece hydrophobic intraocular lenses were inserted into capsular bags. Ocular ointment (tobramycin 0.3% and dexamethasone 0.1%) was topically applied at the end of the surgery. After surgery, all patients used topical prednisolone acetate 1.0%, diclofenac sodium 0.1% and levofloxacin 0.3% eye drops for 4 wks.

### Clinical examinations

All nuclear stages were classified according to LOCS II. Uncorrected visual acuity (UCVA), best corrected visual acuity (BCVA) and intraocular pressure (IOP) were recorded via CT-80A noncontact topography (Topcon Corporation, Tokyo, Japan) of each patient at preoperative, 1-day, 1-week and 3-month postoperative visits.

Central corneal thickness (CCT), anterior chamber depth (ACD) and axial length (AL) were recorded, and intraocular lens diopter was calculated via an OA2000 optical biometer (Tomey, Nagoya, Japan) at preoperative, 1-day postoperative and 1-month postoperative visits.

Endothelial cell density (ECD) was recorded at preoperative and 3-month postoperative visits via Topcon SP3000P noncontact endothelial specular microscopy (Topcon Corporation, Tokyo, Japan). Topcon 3D-OCT-2000 spectral optical coherence tomography (Topcon Corporation, Tokyo, Japan) was used to rule out fundus abnormalities.

Intraoperative energy parameters, including cumulative dissipated energy (CDE), total US time, total aspiration time, total estimated fluid aspirated, average torsional amplitude (ATA), ATA when the foot pad was on gear 3 (ATA-FP3), total torsional amplitude on time, equivalent average torsional amplitude-FP3 (EATA-FP3), average longitudinal power (ALP), average longitudinal power-FP3 (ALP-FP3), total longitudinal power on time and equivalent average ultrasonic power-FP3 (EAUP-FP3), were recorded. According to the definition, CDE = time of torsional power × 0.4 × ATA + time of longitudinal power × ALP, and we recognized the torsional part (time of torsional power × 0.4 × ATA) as cumulative torsional energy (CLE) and the longitudinal part (time of longitudinal power × ALP) as cumulative longitudinal energy (CLE).

### Statistical analysis

All data were analysed using IBM SPSS 26.0 for Mac (IBM Corp., Armonk, NY, USA). Independent t tests were used to analyse the differences in age, energy parameters, CCT, change in CCT and 3-month postoperative ECD between the two groups. The chi-square test was used to analyse the difference in the ratio of sex and eye between the two groups. Pearson correlation analysis was used to analyse the correlation of ACD, total CDE, CTE, CLE, change in 1-day postoperative CCT and change in the 3-month postoperative ECD loss rate. *P* < 0.05 was considered statistically significant.

## Results

A total of 104 eyes of 79 patients were enrolled in this study, and baseline parameters are shown in Table 1. Sex, age, eye, ACD, CCT, ECD and distribution of stage of nucleus between the two groups showed no significant difference. No patient experienced posterior capsule rupture during the surgery or other severe postoperative complications, such as toxic anterior segment syndrome (TASS) or endophthalmitis. UCVA, BCVA and NCT at each follow-up visit showed no difference between the two groups.

**Table 1:** Comparisons of patient characteristics and preoperative parameters

	Balanced tip group (*n* = 47)	Kelman tip group (*n* = 57)	*P* value
Age	69.57 ± 6.64	70.75 ± 8.91	0.454
Patient number	35	47	
Gender (Male/Female)	18(51.4%)/17(48.6%)	14(29.8%)/33(70.2)	0.067
Eye (right/left)	27(57.4%)/20(42.6%)	30(52.6%)/27(47.4%)	0.694
Stage of Nucleus^a^			
2	15(31.9%)	24(42.1%)	0.598
3	21(44.7%)	20(35.1%)	
4	11(23.4%)	13(22.8%)	
ACD (mm)	3.01 ± 0.43	3.04 ± 0.37	0.697
CCT (μm)	514.3 ± 31.1	527.8 ± 24.8	0.057
ECD (/mm^2^)	2359.5 ± 524.2	2450.6 ± 429.4	0.446

*ACD* anterior chamber depth, *CCT* corneal central thickness, *ECD* endothelial cell density

^a^Nucleus grading by LOCSII

**Fig. 2:**
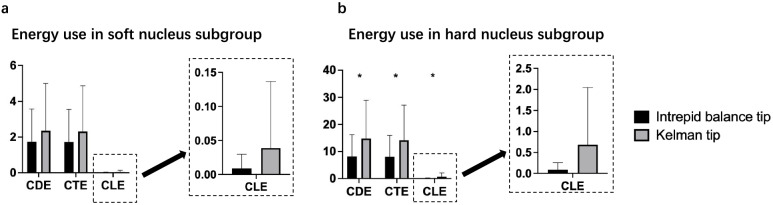
Torsional and longitudinal energy use in different phaco tip groups. **a** In the soft nucleus subgroup, different types of energy use were less common in the balanced tip group than in the Kelman group, without statistical significance. **b** In the hard nucleus subgroup, different types of energy use were significantly higher than those in the soft nucleus subgroup and lower in the balanced tip group than those in the Kelman group (* *p* < 0.05). CDE: cumulative dissipated energy; CTE: cumulated torsional energy; CLE: cumulated longitudinal energy

The total US time, total estimated fluid aspirated, change in CCT and loss rate of ECD at each follow-up visit showed no statistical significance between the IB group and the K group (Table 1). The energy parameters of the IB group, such as ATA, ATA-FP3, EATA-FP3, ALP, ALP-FP3, EAUP-FP3 and CLE, were lower than those of the K group (Table 2). Stratified analysis was carried out according to the stage of the nucleus and classified patients into soft nuclei (stage 2 nucleus) and hard nuclei (stage 3 and 4 nucleus). The total CDE (8.15 ± 8.02 vs 14.82 ± 14.16, *P* = 0.023), CTE (8.06 ± 7.87 vs 14.13 ± 13.02, *P* = 0.027) and CLE (0.09 ± 0.17 vs 0.69 ± 1.37, *P* = 0.017) of the IB group were found to be lower than those of the K group among patients with hard nuclei, but there was no difference among soft nucleus patients (Fig. 2).

The total US time, total estimated fluid aspirated, change in CCT and loss rate of ECD at each follow-up visit showed no significant difference among patients with different stages of nuclei. However, mean change of CCT in IB group(33.0±23.1μm) was smaller than K group(47.4±45.2μm), but owing to huge variants of nucleus and energy used in stage 4 nucleus, there was no statistical significance.

**Table 2:** Comparison of intraoperative parameters and corneal damage in the soft and hard nucleus subgroups

	Total *n* = 104			Soft lens (grade II nucleus) *n* = 39			Hard lens (grade III + IV nucleus) *n* = 65		
	Balanced(*n* = 47)	Kelman(*n* = 57)	*P* value	Balanced(*n* = 15)	Kelman(*n* = 24)	*P* value	Balanced(*n* = 32)	Kelman(*n* = 33)	*P* value
Total case time	561.1 ± 276.5	525.1 ± 389.9	0.596	529.8 ± 282.0	491.2 ± 475.4	0.777	576.1 ± 277.2	549.8 ± 319.4	0.724
total Estimated fluid Aspirated	44.0 ± 20.9	48.5 ± 32.7	0.418	33.9 ± 8.4	31.5 ± 8.5	0.394	48.8 ± 23.3	60.8 ± 37.9	0.127
CDE	6.10 ± 7.32	9.57 ± 12.49	0.081	1.73 ± 1.84	2.35 ± 2.64	0.432	8.15 ± 8.02	14.82 ± 14.16	**0.023**
ATA (%)	27.59 ± 12.26	53.75 ± 13.00	** < 0.001**	17.79 ± 8.60	45.70 ± 14.11	** < 0.001**	32.18 ± 11.03	59.59 ± 8.30	** < 0.001**
ATA-FP3	27.20 ± 12.18	52.80 ± 12.69	** < 0.001**	17.72 ± 8.54	45.40 ± 14.05	** < 0.001**	32.03 ± 10.94	58.19 ± 8.31	** < 0.001**
EATA-FP3	10.88 ± 4.87	21.12 ± 5.07	** < 0.001**	7.09 ± 3.41	18.16 ± 5.62	** < 0.001**	12.81 ± 4.38	23.27 ± 3.32	** < 0.001**
ALP (%)	7.61 ± 11.87	38.13 ± 12.54	** < 0.001**	3.95 ± 7.34	32.27 ± 13.78	** < 0.001**	9.32 ± 13.23	42.39 ± 9.71	** < 0.001**
ALP-FP3	0.08 ± 0.11	0.56 ± 0.50	** < 0.001**	0.04 ± 0.06	0.24 ± 0.17	** < 0.001**	0.09 ± 0.13	0.79 ± 0.53	** < 0.001**
EAUP-FP3	1096 ± 4.92	21.68 ± 5.25	** < 0.001**	7.13 ± 3.44	18.40 ± 5.66	** < 0.001**	12.90 ± 4.43	24.06 ± 3.35	** < 0.001**
CTE	6.04 ± 7.19	9.16 ± 11.58	0.096	1.73 ± 1.82	2.32 ± 2.56	0.442	8.06 ± 7.87	14.14 ± 13.02	**0.027**
CLE	0.06 ± 0.14	0.41 ± 1.08	**0.018**	0.01 ± 0.02	0.04 ± 0.10	0.255	0.09 ± 0.17	0.69 ± 1.37	**0.017**
Change of P1D CCT (μm)	28.96 ± 22.06	35.83 ± 37.84	0.252	20.40 ± 17.25	19.96 ± 14.00	0.931	32.97 ± 23.14	47.36 ± 45.16	0.111
Change of P1W CCT (μm)	6.69 ± 18.57	8.73 ± 16.21	0.559	-0.31 ± 11.59	3.74 ± 18.27	0.477	9.53 ± 20.21	12.31 ± 13.76	0.522
Loss rate of ECD (%)	0.13 ± 0.11	0.11 ± 0.15	0.360	0.07 ± 0.14	0.05 ± 0.12	0.118	0.13 ± 0.13	0.15 ± 0.16	0.715

*CDE* cumulative dissipated energy, *ATA* average torsional amplitude, *ATA-FP3* ATA when the foot pad was on gear 3, *EATA-FP3* total torsional amplitude on time, equivalent average torsional amplitude-FP3, *ALP* average longitudinal power, *ALP-FP3* average longitudinal power-FP3, *EAUP-FP3* total longitudinal power on time and equivalent average ultrasonic power-FP3, *CTE* cumulative torsional energy, *CLE* cumulative longitudinal energy, *CCT* corneal central thickness, *ECD* endothelial cell density, *P1D* one day after surgery, *P1W* one week after surgery

In addition, bivariate correlation analysis was used to analyse factors associated with corneal edema and the loss rate of ECD. The results showed that the loss rate of ECD was negatively correlated with ACD and positively correlated with CDE, CTE, CLE and changes in CCT on the 1-day postoperative visit. In addition, changes in CCT on the 1-day postoperative visit were positively correlated with CDE, CTE and CLE and negatively correlated with ACD (Table 3). We separately analysed the above parameters among different stages of the nucleus and found the same results among patients with hard nuclei, but patients with soft nuclei showed no statistically significant result.

**Table 3:** Bivariate correlation analysis of influencing factors on post-phacoemulsification corneal damage

	Change of P1D CCT						Loss rate of ECD					
	Total		Soft nucleus		Hard nucleus		Total		Soft nucleus		Hard nucleus	
	Correlation coefficient	*P* value	Correlation coefficient	*P* value	Correlation coefficient	*P* value	Correlation coefficient	*P* value	Correlation coefficient	*P* value	Correlation coefficient	*P* value
ACD	-0.124	0.211	-0.077	0.643	-0.18	0.151	-0.259	**0.024**	-0.027	0.887	-0.362	**0.014**
Total CDE	0.519	** < 0.001**	0.084	0.609	0.473	** < 0.001**	0.445	0.088	0.118	0.526	0.473	**0.001**
CLE	0.316	**0.001**	-0.02	0.905	0.283	**0.022**	0.359	**0.001**	0.11	0.556	0.389	**0.008**
CTE	0.526	** < 0.001**	0.088	0.596	0.481	** < 0.001**	0.446	** < 0.001**	0.118	0.526	0.474	0.001

*CCT* corneal central thickness, *ECD* endothelial cell density, *ACD* anterior chamber depth, *CDE* cumulative dissipated energy, *CTE* cumulated torsional energy, *CLE* cumulated longitudinal energy, *P1D* one day after surgery

## Discussion

During the cataract phacoemulsification procedure, phaco tips affect energy efficiency and safety through their energy output, holdability, nucleus fragment followability, and surge suppression. In this study, we focused on the tip efficiency, heat burns and mechanical damage to the cornea caused by phaco tips.

Around the incision area, the needle vibrates with the infusion sleeve to produce a frictional thermal effect, resulting in tissue burns. Under the traditional longitudinal US mode, the movement of the needle is along the longitudinal axis and therefore generates more heat at the incision site and lowers the energy efficiency [1]. Torsional power was first introduced in 2005 and was developed to improve efficiency and decrease energy and fluid use [4–6]. The phaco needle for the torsional mode has one or two inclinations at the distal end; when the needle rotates around the longitudinal axis, a larger amplitude is generated at the distal end of the needle. At the near end of the needle around the incision, only a small displacement is generated. The friction and incision burn are greatly reduced, and the energy efficiency is increased significantly [6].

The phaco tip applied in torsional phacoemulsification includes a shaft and a cutting edge that has one or two bends. Stroke amplification depends on the angle of the bend near the distal end of the needle and the length of the shaft beyond the bend [7].

The balanced tip has a maximum amplitude of 192 μm, which may produce a more efficient US effect with the same energy parameter setting [8, 9]. The cutting efficiency is dependent on both the amplitude and the bevel of the tips. In this study, we selected 45° bevel tips for both groups to compare the efficiency of the needles. The results show that the longitudinal and torsional energy and total CDE used in the IB group are lower than those used in the K group, which confirms their high energy efficiency. This difference was more pronounced in the hard nucleus subgroup and less pronounced in the soft nucleus subgroup.

For hard nucleus phacoemulsification, the phaco needle is often blocked, at which time the phaco energy is continuously released but no nucleus material is crushed, and the energy efficiency is significantly reduced. If the blocked nucleus fragment is pushed away by the tip’s vibration and brought back to the tip opening by the fluid flow, the nucleus material can be emulsified, and the energy efficiency can be increased. The phaco platform offers pulse, burst, and continuous modes. The IP software delivers alternating torsional and longitudinal pulses after a preset maximum vacuum level (95% in this study for both groups) is reached, which reduces tip clogging during emulsification of hard cataracts. All the longitudinal energy used in this study came from pulses in IP mode. More blocking occurrences and a longer blocking time during phacoemulsification will trigger more longitudinal energy and a longer use time. The results show that the CLE of Group K is statistically higher than that of Group IB, indicating that the balanced needle is less likely to cause obstruction due to its large swing, suggesting that the larger amplitude of the balanced needle is more efficient for the hard nucleus. This advantage is not significant in the soft nucleus subgroup, as soft nucleus US rarely causes needle blocking and longitudinal energy is rarely triggered.

The increase in CCT on the first postoperative day and corneal endothelial loss rate at 3 months are indicators of short-term and long-term corneal damage from cataract surgery. The damage mainly comes from the mechanical damage caused by the turbulence of the aqueous humor, the impact of the nuclear fragment on the corneal endothelium and the incision burns caused by phaco needle vibration. Tissue burns can be avoided by decreasing heat production and sufficient infusion in the irrigation sleeve, which can cool down the needle. The Kelman needle is paired with a standard infusion sleeve that matches for a 3.2 mm corneal incision, while the balanced needle is paired with an ultra-perfusion sleeve, which is suitable for a 2.2 mm corneal incision. Asmaller incision and irrigation sleeve will theoretically limit the infusion flow. Previous studies have included balanced needles with an active fluidics infusion system to maintain sufficient infusion and IOP [10, 11]. To our knowledge, a comparative study of balanced needles with gravity perfusion systems with Kelman needles under the same system has not been reported. The results of this study showed no significant difference in either US time and estimated fluid volume or the corneal endothelium loss rate at 3 months postoperatively between the two groups, implying the noninferiority of the balanced needle compared with the Kelman needle in terms of intraocular tissue damage, which also indicates that the ultra-perfusion sleeve can provide sufficient perfusion flow within a 2.2 mm incision.

According to the two-variable correlation analysis in the study, postoperative corneal edema and endothelial cell loss were positively correlated with the total energy used in surgery, as well as longitudinal and torsional energy use. Lower energy use may create less frictional heat at the incision site, which reduces incision burns and causes less mechanical tissue damage. In this study, lower CCT changes in the IB group on the first postoperative day were observed without statistical significance. Further analysis of the original data reveals that when the nucleus is hard and more energy is used, the standard deviation of CCT changes increases considerably, which will affect the statistical results. Increasing the sample size will help to achieve a more convincing result. ACD should also be considered when evaluating corneal damage.

Jensen et al. observed the in vitro efficiency of a balanced phaco tip on the Centurion® system and reported a linear relationship of increasing efficiency correlating with increasing power from a 30% to 60% power level. No further improvement in efficiency was observed above 60% power [12]. In the preexperiment of our study, we used the same power (50% to 85%) in the two groups and found more obvious corneal edema in the IB group on the first postoperative day. Then, we adjusted the power level of the IB group to 30%-60% according to previous studies. Since the amplitude of the balanced needle (192 μm) is 48% higher than that of the Kelman needle (130 μm), the same energy setting will generate different amplitudes at the distal ends of the needles in the two groups. A balanced tip is more likely to present higher efficiency and more corneal damage, as shown in previous literature [8, 13, 14]. To achieve the same amplitude as balanced needles, we set the energy from 50 to 85% for the Kelman group in our study. We expect comparable corneal damage in the two groups under such energy settings, which is in accordance with our results.

Postoperative CCT and ECD loss are affected not only by the efficacy of the phaco tips but also by the corneal incision size and the perfusion sleeves, which are different in each group. The results of the study showed total differences between the two group settings. The corneal incision size and the perfusion sleeve effects were not analysed. A larger sample size will help to reach more convincing conclusions.

## Conclusions

In summary, a balanced phaco needle is an energy-efficient needle covered by an ultra-perfusion sleeve within a 2.2 mm corneal incision to complete cataract phacoemulsification with a lower energy setting without leading to additional short-term and long-term corneal damage. The advantages of the needle are more pronounced in hard nuclear cataracts, and needle blocking occurs less frequently.

## Data Availability

The datasets generated and/or analysed during the current study are not publicly available due to limitations of ethical approval involving the patient data and anonymity but are available from the corresponding author on reasonable request.
